# Fisetin inhibits the proliferation, migration and invasion of pancreatic cancer by targeting PI3K/AKT/mTOR signaling

**DOI:** 10.18632/aging.203713

**Published:** 2021-11-25

**Authors:** Yanyi Xiao, Yilong Liu, Zhiwei Gao, Xian Li, Min Weng, Chenghao Shi, Cheng Wang, Linxiao Sun

**Affiliations:** 1 Key Laboratory of Diagnosis and Treatment of Severe Hepato-Pancreatic Diseases of Zhejiang Province, Zhejiang Provincial Top Key Discipline in Surgery, Wenzhou Medical University First Affiliated Hospital, Wenzhou 325015, Zhejiang, China; 2 Zhejiang Provincial Key Laboratory of Horticultural Plant Integrative Biology, The State Agriculture Ministry Laboratory of Horticultural Plant Growth, Development and Quality Improvement, Zhejiang University, Hangzhou 310058, Zhejiang, China; 3 School of Medicine, The Second Affiliated Hospital of Zhejiang University, Hangzhou 310009, Zhejiang, China

**Keywords:** fisetin, apoptosis, PI3K/AKT/mTOR, pancreatic cancer, proliferation

## Abstract

Pancreatic cancer is an extremely malignant digestive tract tumor. With the increase of chemotherapeutic resistance of pancreatic cancer, clinical treatment is in a dilemma. Hence, it is pivotal to design an effective drug for treating individuals with pancreatic cancer. Fisetin extracted from vegetables, as well as fruits was explored to possess antioxidant, anti-cancer, anti-inflammatory along with anti-microbial properties. Nonetheless, there is limited research focusing on the utility of fisetin as an inhibitor of pancreatic cancer. Similarly, the mechanism through which Fisetin dampens pancreatic cancer remains unknown. This research work systematically evaluated the possible anti-cancer influences of fisetin in pancreatic cancer, as well as explored its responsible molecular mechanism. Our data revealed that fisetin obviously dampens pancreatic cancer progress *in vitro* along with *in vivo* dose-dependently. Furthermore, we established that fisetin repressed pancreatic cancer via explicitly targeting PI3K/AKT/mTOR signaling cascade and not the JAK2 cascade. Our data clarified that fisetin is a prospective anti-cancer drug for pancreatic cancer, as well as indicated the distinct molecular target of fisetin.

## INTRODUCTION

Pancreatic cancer (PC) is a fatal malignancy with a median survival time of six months after diagnosis and a five-year overall survival (OS) of only 5% [1, 2]. It is dominated by pancreatic ductal adenocarcinoma, a most common type accounting for approximately 95% of all PCs [3, 4]. At present, surgery is known as the most effective way for the treatment of PC. In contrast, only less than 20% can receive surgical resection since most patients have already reached an advanced stage once diagnosed [5]. In addition, adjuvant chemotherapy may improve the condition or expand the life of PC patients who are not suitable for curative surgery. However, it causes severe side effects on patients, such as constipation, loss of appetite, vomiting, nausea, etc. [6, 7]. Hence, it is urgently needed to design new drugs with good effects and low toxicity for the control or treatment of PC.

In the last few decades, numerous studies have shown that various natural phytochemicals, such as plant-derived alkaloids and flavonoids, exhibited significant anti-cancer activities [8]. Also, many first-line anti-cancer drugs are derived from natural products, such as paclitaxel and colchicine. Hence, the exploration of natural agents against numerous human cancers has attracted the attention of the scientific research [9]. Compounds extracted from plants belonging to diverse groups, for instance as alkaloids, flavonoids, as well as polyphenols, were explored for their anti-cancer influences, and potential data were obtained, thus providing a possible treatment approach for some human cancers [10]. Through the screening of these natural products, we have found that some natural products have strong anti-pancreatic cancer effects, such as Baohuoside 1 and betulinic acid, which may become effective agents for treating pancreatic cancer [11, 12]. Fisetin (3,3’,4’,7-tetrahydroxyflavone), which distributes abundantly in vegetables, as well as fruits for instance cucumber, persimmon, and apple, was explored to possess antioxidant, anti-microbial, anti-cancer, as well as anti-inflammatory properties in recent studies [13]. For example, fisetin could dampen colorectal cancer cells growth *in vitro* along with *in vivo* [14] and display anti-cancer effects on laryngeal carcinoma [15]. However, there is no report about the anti-cancer influence of fisetin on PC.

Herein, we investigated the *in vitro* anti-cancer influence of fisetin on the growth, invasion along with migration of PANC-1, as well as Patu-8988 cells. We evaluated the *in vivo* effect on PC by using the nude mice as an experimental model. Possible and preliminary mechanisms of such development were also investigated and discussed *in vitro*, as well as *in vivo* and additionally assessed the potential molecular mechanism. Our results suggested that fisetin inhibited proliferation, infiltration along with migration and triggered pancreatic cancer cells apoptosis through targeting the PI3K/AKT/mTOR cascade. Thus, fisetin might be a possible and valuable anti-cancer drug for pancreatic cancer.

## MATERIALS AND METHODS

### Drugs

Astragaline (CAS No. : 480-10-4, purity of 99.85%), Afzelin (CAS No. : 482-39-3, purity of 99.62%), Quercetin 3-O-α-L-arabinoside (CAS No. : 22688-79-5, purity of 99.83%), Narcissoside (CAS No. : 604-80-8), Avicularin (CAS No. : 572-30-5), Fisetin (CAS No. : 528-48-3, purity of 98.02%), Herbacetin (CAS No. : 527-95-7, purity > 99.0%) were purchased from MedChemExpress (MCE, Shanghai, China). 3'-Hydroxyflavanone (CAS No. : 1621-55-2), Kaempferol-3-O-rutinoside (CAS No. : 17650-84-9, purity ≥ 98%) were obtained from YuanYe Biotechnology (Shanghai, China).

### Cells culture and drug treatment

The PANC-1 along with the Patu-8988 human pancreatic cells were acquired from the Cell Bank of the Chinese Academy of Sciences (Shanghai, China). PANC-1 cells and Patu-8988 cells were inoculated in DMEM (Invitrogen, CA, United States) added with FBS (10%; Invitrogen), streptomycin (100 μg ml^-1^) along with penicillin (100 U ml^-1^). 1 × 10^6^ PANC-1 cells and 1 × 10^6^ Patu-8988 cells were planted for 24 hours at 37° C. Afterwards, complete medium was changed with a new cultural medium prior to inoculation of the cells with fisetin.

### Real-time cellular analysis (RTCA)

2.5 × 10^4^ PANC-1 cells were inoculated in the E16-culture plate (ACEA Biosciences, United States), and then RTCA (Roche, Germany) was adopted to document the cellular growth index automatically.

### CCK-8 assay

The CCK-8 Kit (Biosharp, BS350B) was employed to explored viability of HPNE, PANC-1, as well as Patu-8988 cells inoculated with fisetin (160 μM, 140 μM, 120 μM, 100 μM, 80 μM, 60 μM, 40 μM, 20 μM, and 0 μM) and allowed to grow for 24 hours. After that, we introduced 10 μL of the reagent to the well harboring 100 μL of 5 × 10^3^ cell suspensions incubated for one hour, and OD read at 450 nm. Cell viability percentage was computed via comparison with the control cells (untreated).

### Flow cytometry analysis

Annexin V-FITC Apoptosis Detection Kit I was supplied by BD Pharmingen™. PANC-1 cells along with the Patu-8988 cells were inoculated in complete medium with diverse levels of fisetin for 24 hours. For apoptosis analysis, cells were collected by spinning and re-suspended in binding buffer. Annexin V-FITC was inoculated in the dark with the resuspended cells for 15-20 minutes at RT (room temperature). Afterwards, we added propidium iodide (PI) into resuspended cells for another 5-10 minutes in the dark. At last, cells apoptosis was assessed on flow cytometry (BD FACSVerse™, BD Biosciences, USA).

### Transwell invasion assay

The transwell invasion assay was adopted to explore the infiltration potential of PANC-1, as well as Patu-8988 cells. Coating of the transwell inserts with 100 μl Matrigel was done for four hours at 37° C. PANC-1 cells along with the Patu-8988 cells were harvested and then re-suspended in serum-free DMEM. 5 × 10^4^ cells/well were planted in the upper compartment, and the lower compartment was added with 500 μl DMEM enriched with 10% FBS. Thereafter, we incubated the samples at 37° C for 24 hours, followed by removal of the gel along with the cells in the upper compartment. Thereafter formalin fixation was done, and then PBS was used to clean 2 times. Next, staining of cells (in crystal violet) was performed for 15 minutes. Lastly, the numbers of infiltrated cells in five random selected fields were determined under a microscope (Leica Microsystems, Germany).

### Colony formation assay

1000-2500 PANC-1 cells/well and 1000-2500 Patu-8988 cells/well were planted in 6-well plates for 24 hours, and then were inoculated with different concentration of fisetin for another 24 hours, fresh DMEM was used to replace the culture medium and continue to culture for 14 days. Fixation of colonies with formaldehyde was done for 30 minutes and then cleaned 2 times with PBS. Crystal violet was employed to stain the colonies. Finally, the number of colonies were counted.

### Wound healing assay

PANC-1 cells, as well as Patu-8988 cells were inoculated in 6-well plates 37° C for 24 hours to make cells grow to cover the plate. Then, scratching of the culture area was done with a crystal pipette tip to make a linear gap among the cells. Next, PBS was utilized to wash away the detached cells and then introduced different concentration of fisetin. Finally, the cells were to grow for 24 hours to fill the gap, and then images were acquired with a microscope (Leica Microsystems, Germany).

### Immunocytochemical staining

Immunofluorescence staining was carried out as documented previously. Firstly, PANC-1 cells along with Patu-8988 cells were inoculated with diverse levels of fisetin and left to grow on glass coverslips for 24 hours. Thereafter, fixation of cells (in 4% formaldehyde) was done for 30 minutes. Afterwards, the cells were infiltrated with Triton X-100 (0.1%), and then blocked the PANC-1 cells with normal goat serum (4%) for one hour. Next, PANC-1 cells, as well as Patu-8988 cells were overnight inoculated with primary antibody against Ki67 (ab15580; 1:200; Abcam) at 4° C, and the inoculation with secondary antibodies was done at 37° C (1:400; Santa Cruz Biotechnology, USA) for 1.5 h. Finally, DAPI (Beyotime Biotechnology, Shanghai, China) was used to stain the cells, and then images were captured under a fluorescence microscope.

### Western blot analysis

Collected whole cellular proteins and then determined the protein concentrations by BCA. Next, 45 μg proteins was resolved over 10% polyacrylamide gels and then transfer-embedded onto a PVDF membrane (Solarbio, Beijing, China). Blocking of the membrane was done in 5% non-fat milk for two hours at RT and then overnight inoculated with the appropriate primary antibodies at 4° C. For PI3K/AKT/mTOR pathway, anti-PI3K (ABclonal; 1:1000), anti-AKT (ABclonal; 1:1000), anti-p-AKT (Abcam; 1:1000), anti-mTOR (Abcam; 1:1000) and anti-p-mTOR (Abcam; 1:1000) antibodies were used. For cellular apoptosis, anti-c-PARP (1:1000, Proteintech), anti-caspase-3 (Proteintech, Wuhan, China; 1:1000) and anti-caspase-8 (Proteintech; 1:1000) antibodies were used. For EMT-related proteins, anti-N-cadherin (Abcam; 1:1000), anti-E-cadherin (ProteinTech; 1:1000), anti-α-SMA (Affinity Biosciences; 1:1000), anti-Vimentin (Affinity Biosciences; 1:1000), anti-Collagen I (Abcam; 1:1000) and anti-Collagen III (Abcam; 1:1000) were used. GADPH (Bioworld; 1:1000) was employed as the internal reference. After rinsing thrice in TBST, incubated the membranes with anti-rabbit secondary antibody (Proteintech; 1:5000) for one hour at RT. Lastly, the protein bands were rinsed five times in TBST and visualization via chemiluminescence detection done on the autoradiographic film.

### Nude mouse tumorigenicity assay

Twelve (six to eight weeks old) male nude mice (BALB/c) with 18-22 g weight were obtained from the Experimental Animal Centre of Wenzhou Medical University (Wenzhou, China). All the study mice were reared at temperature-, light-, as well as humidity-controlled conditions, and fed on a standard mice chow and water. The right hind limb of nude mice (n = 12) was inoculated subcutaneously with 3 × 10^6^ PANC-1 cells in 100 μl PBS, then study group mice (n=6) were given intragastric inoculation of fisetin (35 mg/kg) every three day for 30 days and control mice (n = 6) received intragastric administration of solvent (DMSO). Tumors were monitored daily for 30 days. Tumor volumes were measured every five days based on the formula V = (width^2^×length) /2. After the 30 days, we sacrificed these mice with 100% carbon dioxide to detect tumor formation [16]. The Institutional Animal Care and Use Committee of Wenzhou Medical University, China granted approval of the study. The protocols were as per the guidelines of the Institutional Review Board of Wenzhou Key Laboratory of Surgery, China.

### Statistical analysis

The data are given as the means ± standard deviations. Results were analyzed using GraphPad Prism 6.02 Software. A two-tailed Student’s *t*-test was employed to determine statistical significance, with *P*<.05 signifying statistical significance. All experiments were repeated at least 3 times.

### Ethics approval and consent to participate

The animal study protocols, including the method involving animals euthanasia were approved by the Institutional Animal Care and Use Committee of Wenzhou Medical University, China. The methods were also performed according to the guidelines approved by the Institutional Review Board of Wenzhou Key Laboratory of Surgery, China.

## RESULTS

### Fisetin dampens the proliferation of pancreatic cancer by downregulating Ki67 expression

**Figure 1:**
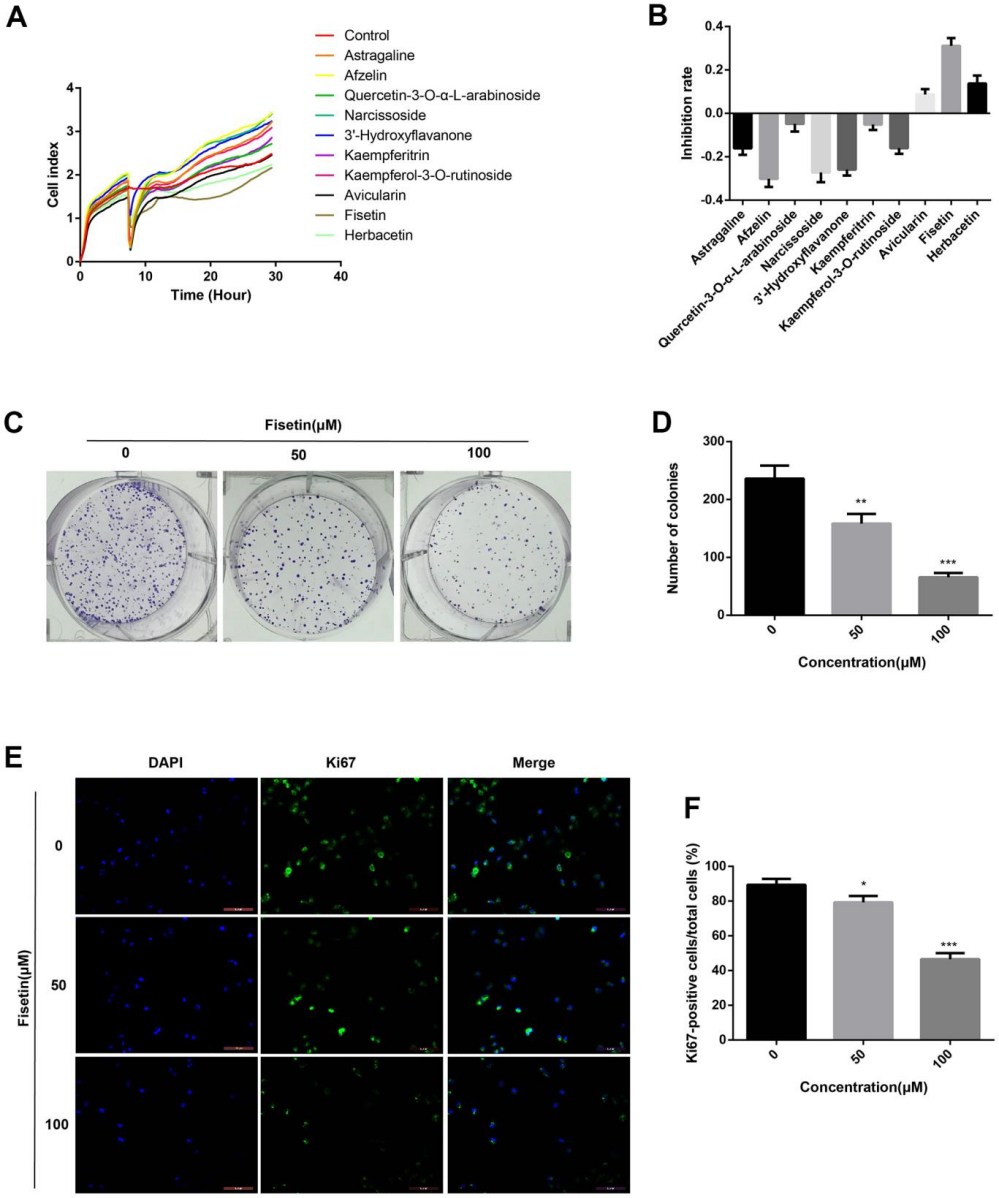
**Fisetin inhibits the proliferation of PANC-1 cells.** (**A**) PANC-1 cells were inoculated with Astragaline, Afzelin, Quercetin 3-O-α-L-arabinoside, Narcissoside, Avicularin, Fisetin, Herbacetin, 3'-Hydroxyflavanone and Kaempferol-3-O-rutinoside (50 μM), and the cell index was recorded by RTCA. (**B**) Histogram exhibiting the repression rate of different compounds. (**C**) Colony formation assessment of PANC-1 cells inoculated with 0,50 and 100 μM fisetin. (**D**) Histogram illustrating the number of colonies in each group. (**E**) Ki67 Immunofluorescence staining of PANC-1 cells inoculated with 0,50 and 100 μM fisetin for 24h. (**F**) Histogram illustrating the Ki67 positive rate of PANC-1 cells in each group. All assays were replicated thrice, and data are given as means±SD.*p<.05,**p<.01,***p<.001, in contrast with the controls.

**Figure 2:**
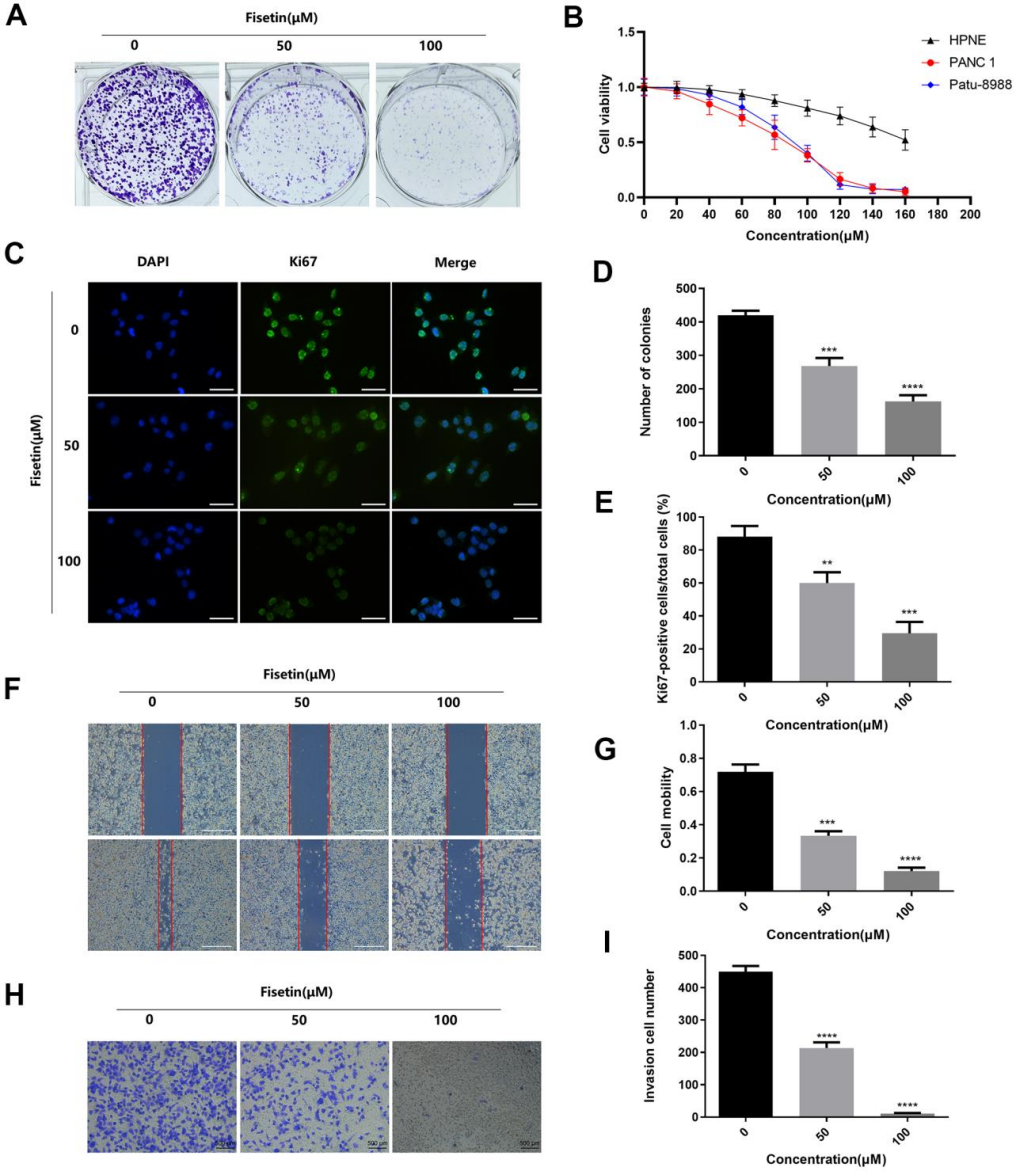
**Fisetin dampens the proliferation, migration along with infiltration of Patu-8988 cells.** (**A**) Colony formation assessment of Patu-8988 cells inoculated with 0,50 and 100 μM fisetin. (**B**) CCK-8 assay of the HPNE, PANC-1 and Patu-8988 cells inoculated with 160 μM, 140 μM, 120 μM, 100 μM, 80 μM, 60 μM, 40 μM, and 20 μM of Fisetin or an equivalent DMEM medium volume for 48 h. (**C**) Ki67 Immunofluorescence staining of Patu-8988 cells inoculated with 0,50 and 100 μM fisetin for 24h. Bar = 50μm. (**D**) Histogram representing the number of colonies in each group. (**E**) Histogram illustrating Ki67 positive rate of Patu-8988 cells in every group. (**F**) Wound healing assays of Patu-8988 cells inoculated with 0, 50 and 100μM fisetin for 24 h. Bar = 500μm. (**G**) Histogram illustrating cell mobility of Patu-8988 cells in every group. (**H**) Transwell assays of Patu-8988 cells inoculated with 0, 50 and 100μM fisetin for 24 h. (**I**) Histogram illustrating the invasion cell number in every group. All experiments were replicated thrice, and data are given as means±SD.*p<.05, **p<.01, ***p<.001, ****p<.0001, in contrast with the controls.

To detect the effects of Astragaline, Afzelin, Quercetin 3-O-α-L-arabinoside, Narcissoside, Avicularin, Fisetin, Herbacetin, 3'-Hydroxyflavanone and Kaempferol-3-O-rutinoside (50 μM) on the growth of human pancreatic cancer cell (PANC-1), unlabeled real-time cell analysis (RTCA) were carried out. As showed in Figure 1A, 1B, the growth of PANC-1 cells remarkably decreased after the administration of fisetin, Avicularin, and Herbacetin; among them, fisetin had the most potent inhibitory effect. Thus, we chose fisetin for subsequent studies. Next, to detect the long-term effects of fisetin on PANC-1 cells, as well as Patu-8988 cells proliferation, plate colony formation assay was conducted. The number of colonies of PANC-1 cells along with Patu-8988 cells treated with fisetin (50 μM, 100 μM) was considerably lower in contrast with that of the controls (Figures 1C, 1D, 2A, 2B), which shows that fisetin could dampen the growth and clonogenicity of the PANC-1 cells along with Patu-8988 cells. Then, we detected the expression of Ki67, a marker of cell proliferation. Immunofluorescence staining exhibited that proliferation of the PANC-1 cells along with Patu-8988 cells remarkably decreased after treatment with 50 μM or 100 μM fisetin (Figures 1E, 1F, 2C, 2D). All of these results indicate that fisetin can dampen the proliferation of pancreatic cancer cells via downregulating Ki67 expression dose-dependently.

### Fisetin triggers apoptosis of human pancreatic cancer cells

**Figure 3:**
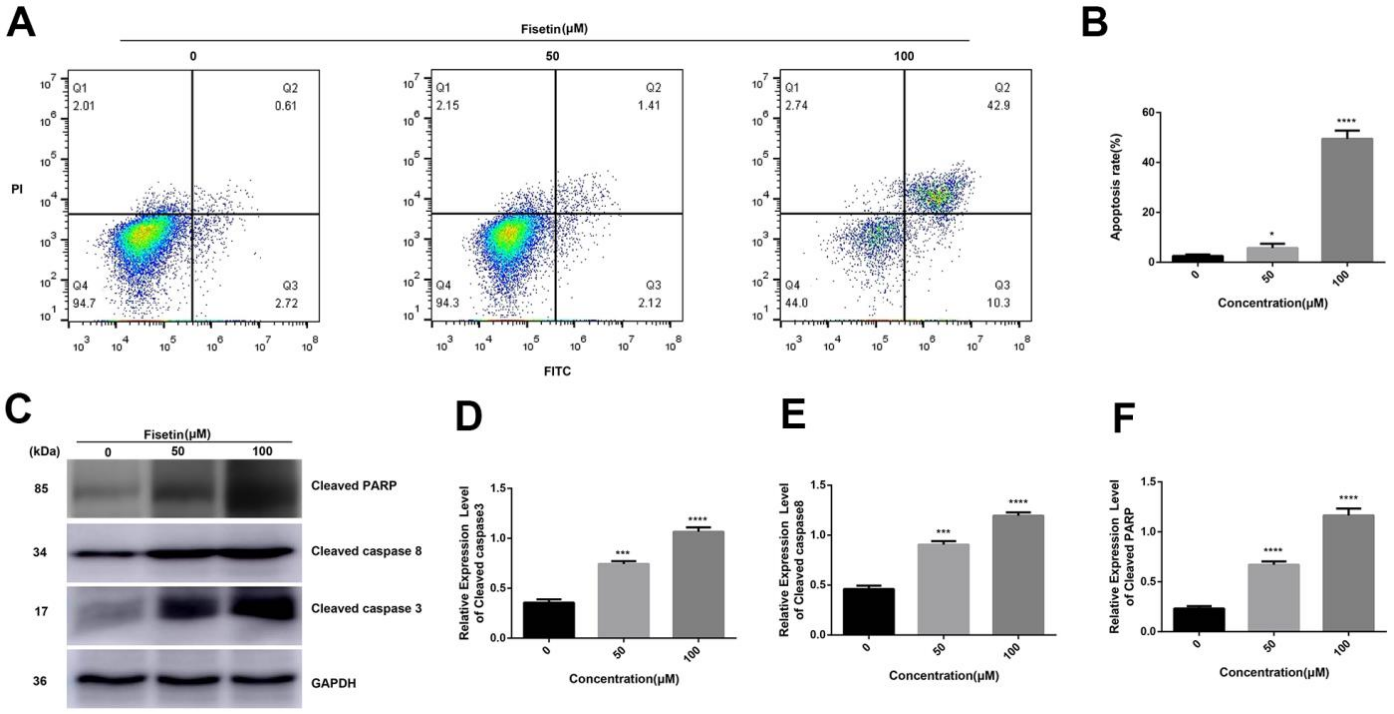
**Fisetin induces the apoptosis of PANC-1 cells.** (**A**) Flow cytometry evaluation of PANC-1 cells inoculated with 0, 50 and 100μM fisetin for 24 h. (**B**) Histogram exhibiting apoptosis rate in each group. (**C**) Western blot assessment of apoptosis-linked proteins. (**D**) Histogram illustrating cleaved caspase 3 protein contents. (**E**) Histogram illustrating cleaved caspase 8 protein contents. (**F**) Histogram illustrating cleaved PARP protein contents. All assays were replicated thrice and data are given as means±SD. *p<.05, ***p<.001, ****P<.0001, in contrast with the controls.

**Figure 4:**
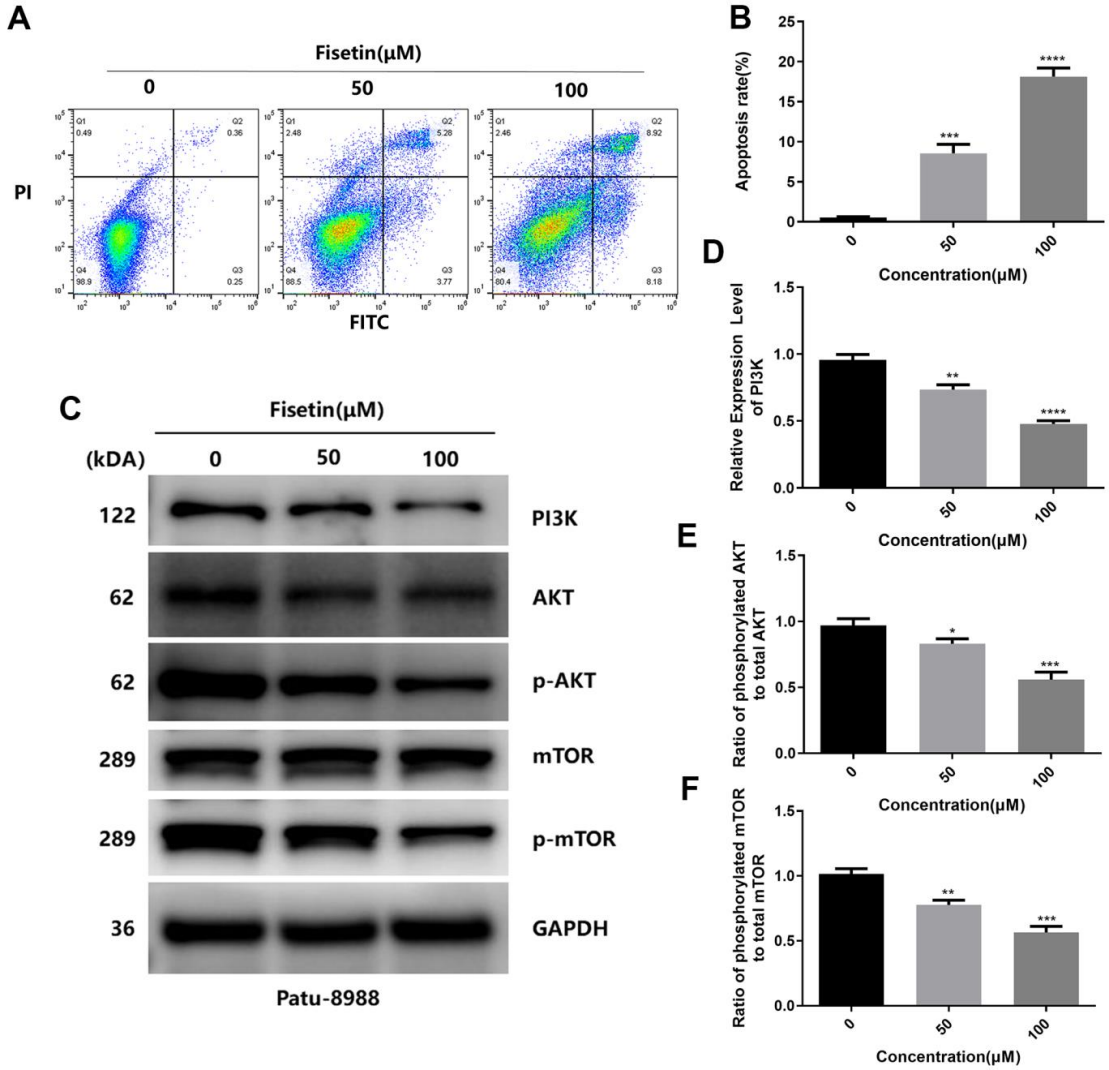
**Fisetin induces the apoptosis and dampens the PI3K/AKT/mTOR cascade in Patu-8988 cells.** (**A**) Flow cytometry evaluation of Patu-8988 cells after inoculation with 0, 50 and 100μM fisetin for 24 h. (**B**) Histogram exhibiting apoptosis rate in each group. (**C**) Western blot assessment of key proteins in Patu-8988 cells inoculated with 0, 50 and 100μM fisetin for 24 h. (**D**) Histogram illustrating PI3K protein contents. (**E**) Histogram illustrating p-AKT/AKT ratio. (**F**) Histogram illustrating p-mTOR/mTOR ration. All assays were replicated thrice, and data are given as means±SD.*p<.05, **p<.01, ***p<.001, ****p<.0001, in contrast with controls.

To detect the influence of fisetin on the apoptosis of human pancreatic cancer cells, AnnexinV-FITC/PI method was performed. As shown in Figures 3A, 3B, 4A, 4B after treatment with 50 μM or 100 μM fisetin for 24 hours, the fraction of pancreatic cancer cells apoptotic cells escalated from 3.33% to 53.20% in contrast with the control group. Meanwhile, the extent of pancreatic cancer cells apoptosis was enhanced as the concentration of fisetin increased. To assess the potential mechanism of fisetin-triggered apoptosis in pancreatic cancer cells, we determined apoptosis-linked proteins’ expressions via western blotting. As illustrated in Figures 3C–3F, the terms of activated caspase 3 and activated caspase 8 were drastically increased by the treatment of fisetin. We also found the expression of cleaved PARP was upregulated indicating that fisetin promoted apoptosis of PANC-1 cells through the mitochondrial-dependent cascade dose-dependently.

### Fisetin reduces the ability of infiltration and migration

**Figure 5:**
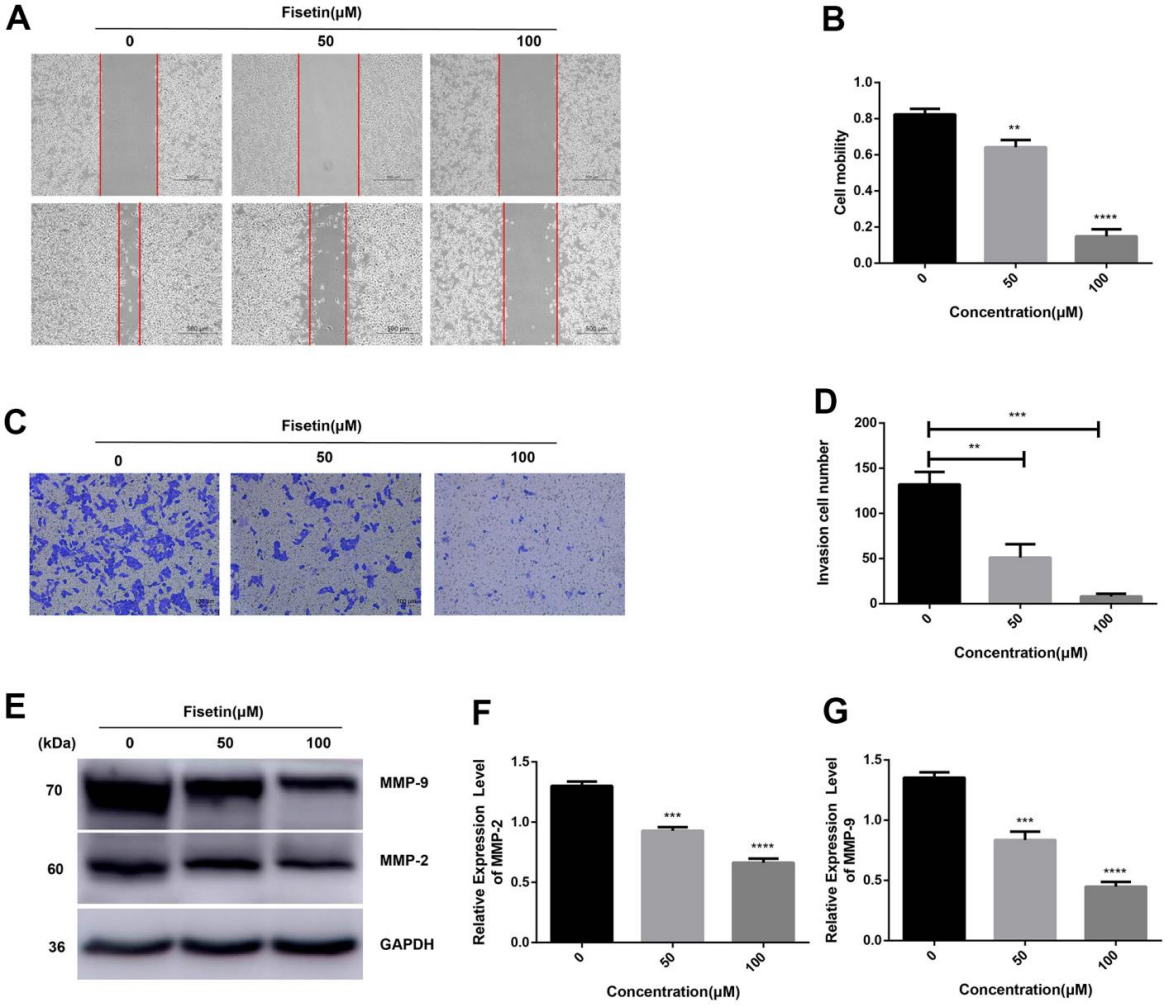
**Fisetin dampens the migration along with the infiltration of PANC-1 cells.** (**A**) Wound healing assays of PANC-1 cells inoculated with 0, 50 and 100μM fisetin for 24 h. (**B**) Histogram exhibiting PANC-1 cell mobility in each group. (**C**) Transwell assays of PANC-1 cells inoculated with 0, 50 and 100μM fisetin for 24 h. (**D**) Histogram illustrating the infiltration cell number in each group. (**E**) Western blot assessment of metastasis-linked proteins. (**F**) Histogram illustrating MMP-2 protein contents. (**G**) Histogram illustrating MMP-9 protein contents. All experiments were replicated thrice and data are given as means±SD. **p<.01, ***p<.001, ****p<.0001, in contrast with the controls.

Next, we detected the ability of cell invasion through wound healing assay. As illustrated in Figure 5A, 5B, 2F, 2G. Fisetin remarkably reduced the migration capacity of Pancreatic cancer cells compared with the untreated group. Furthermore, transwell assay was adopted to determine the influence of fisetin on invasive ability in the PANC-1 cells along with Patu-8988 cells. The result of transwell assay showed that Pancreatic cancer cells treated without fisetin exhibited strong invasive ability, which was obviously reduced in the presence of fisetin (Figures 5C, 5D, 2H, 2I). Furthermore, we revealed that fisetin treatment dampened the expressions of MMP-2 along with MMP-9, which were involved in cellular metastasis (Figure 5E–5G). These data illustrate that fisetin can reduce the ability of infiltration along with migration in pancreatic cancer cells dose-dependently.

### Fisetin dampens the expression of EMT-linked proteins in PANC-1 and Patu-8988 cells

**Figure 6:**
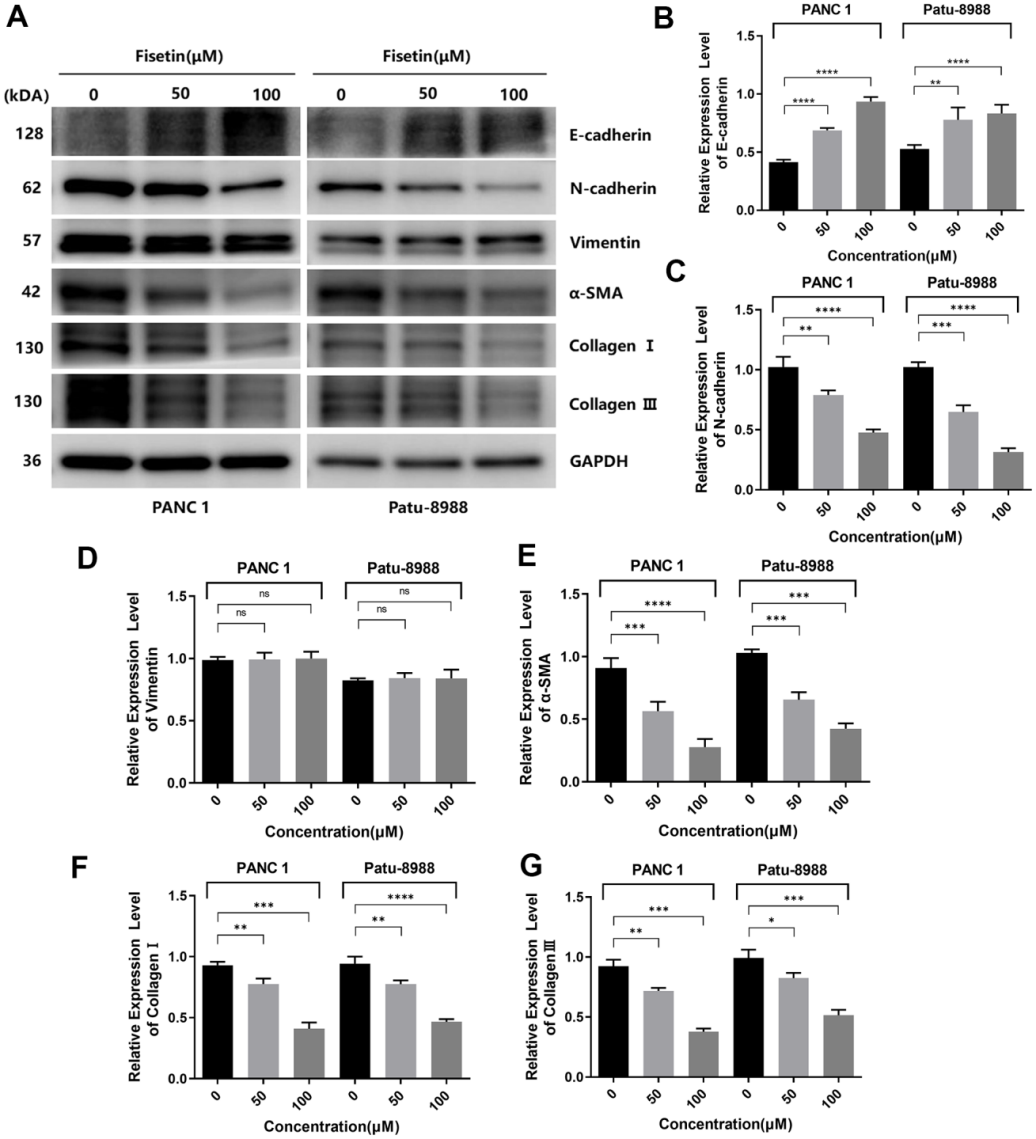
**Fisetin inhibits the expression of EMT-related proteins in PANC-1, as well as Patu-8988 cells.** (**A**) Western blot assessment of EMT-linked proteins in Patu-8988, as well as PANC-1 cells inoculated with 0, 50 and 100μM fisetin for 24 h. (**B**) Histogram illustrating E-cadherin protein contents. (**C**) Histogram illustrating N-cadherin protein contents. (**D**) Histogram illustrating Vimentin protein contents. (**E**) Histogram illustrating alpha-SMA protein contents. (**F**) Histogram illustrating Collagen I protein contents. (**G**) Histogram illustrating Collagen III protein contents. All assays were replicated thrice, and data are given as means±SD.*p<.05, **p<0.01, ***p<.001, ****p<.0001, in contrast with the controls.

Several investigations have pointed out that EMT is crucial in promoting infiltration and migration in tumor cells [17, 18]. Therefore, we explored the influence of fisetin on EMT of different pancreatic cancer cell lines. In PANC-1, as well as Patu-8988 cells, fisetin did not remarkably dampen the content of cytoskeleton-linked protein Vimentin (Figure 6A, 6D). However, we found that fisetin could remarkably elevate the content of epithelial marker E-cadherin and dampened the content of interstitial markers N-cadherin along with a-SMA (Figure 6A–6D), which resulted in the decrease of extracellular matrix (ECM) secreted by stromal tumor cells, for instance type I and III collagen (Figure 6A, 6F, 6G). In addition, we have also observed that the expressions of metalloprotease system linked proteins, consisting of MMP-2 along with MMP-9, which have remarkable modulatory effects on tumor cell invasion and migration, are also inhibited by fisetin (Figure 5E), which proved again fisetin dampened infiltration, as well as migration in pancreatic cancer cells.

### Fisetin dampens the PI3K/AKT/mTOR axis in pancreatic cancer cells

**Figure 7:**
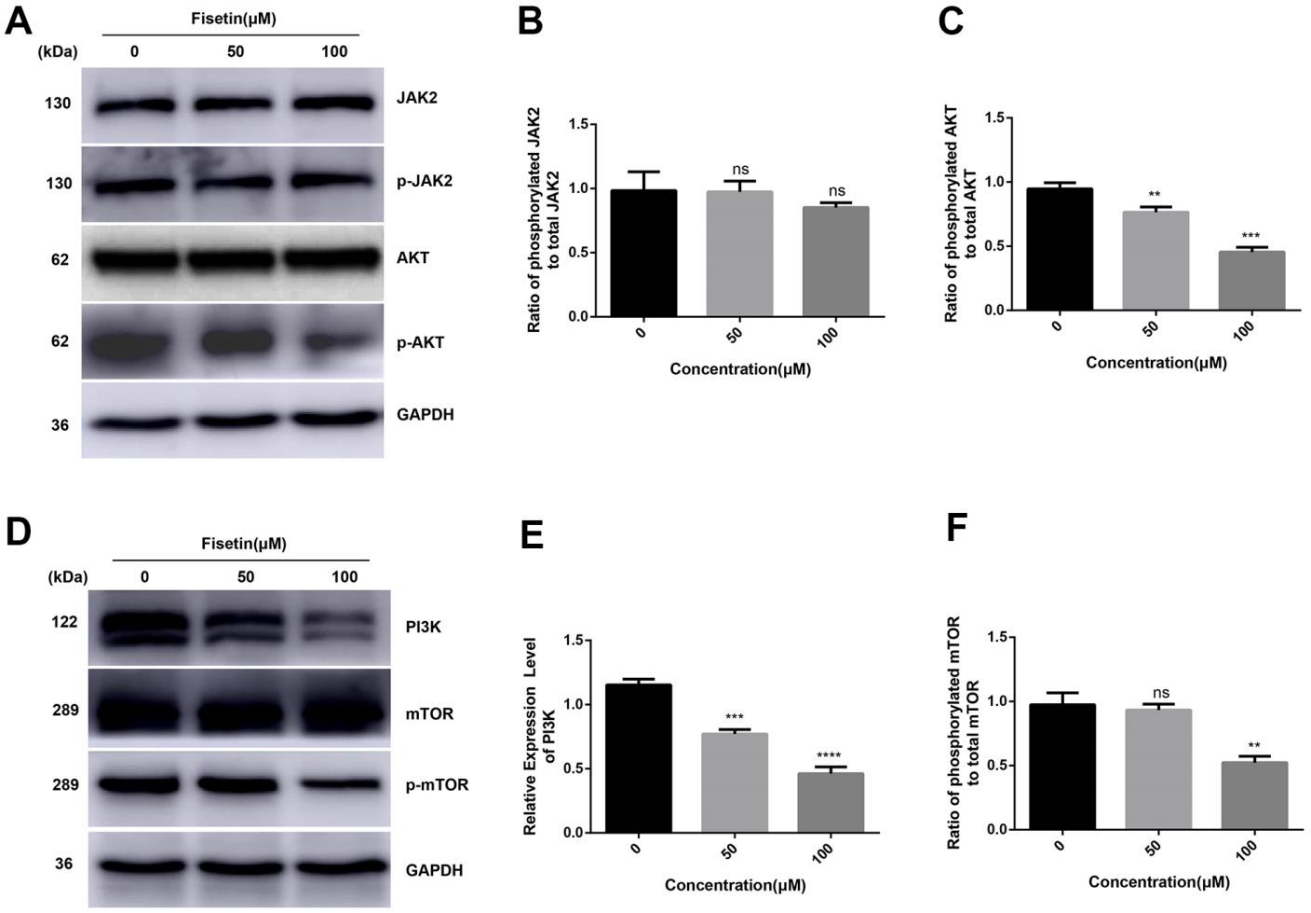
**Fisetin represses the PI3K/AKT/mTOR cascade in PANC-1 cells.** (**A**, **D**) Western blot evaluation of key proteins in PANC-1 cells inoculated with 0, 50 and 100μM fisetin for 24 h. (**B**) Histogram illustrating p-JAK2/JAK2 ratio. (**C**) Histogram illustrating p-AKT/AKT ratio. (**E**) Histogram illustrating PI3K protein content. (**F**) Histogram exhibiting p-mTOR/mTOR ratio. All experiments were replicated thrice and data are given as means±SD. ns P>.05, **P<.01, ***P<.001, ****P<.0001, in contrast with the controls.

As reported, PI3K/AKT/mTOR participates in fisetin-triggered repression in the growth of laryngeal carcinoma cells [15]. Hence, we speculated that fisetin harbors protective influences may by targeting PI3K/AKT/mTOR axis in pancreatic cancer cells. As illustrated in Figures 7A–7C, 4C we observed the expressions of AKT, JAK2 and p-JAK2 proteins in groups inoculated with fisetin had no obvious changes, while p-AKT was downregulated with the control group. Furthermore, we found the total mTOR did not change, nonetheless the p-mTOR and PI3K decreased remarkably (Figures 7D–7F, 4C–4F), indicating that the PI3K/AKT/mTOR cascade participates in inhibitory of PANC-1 cells induced by fisetin. Therefore, we speculate that fisetin may repress the growth, invasion along with the migration of pancreatic cancer cells via dampening the PI3K/AKT/mTOR cascade.

### Fisetin dampens pancreatic tumor growth of cell xenografts in nude mice

**Figure 8:**
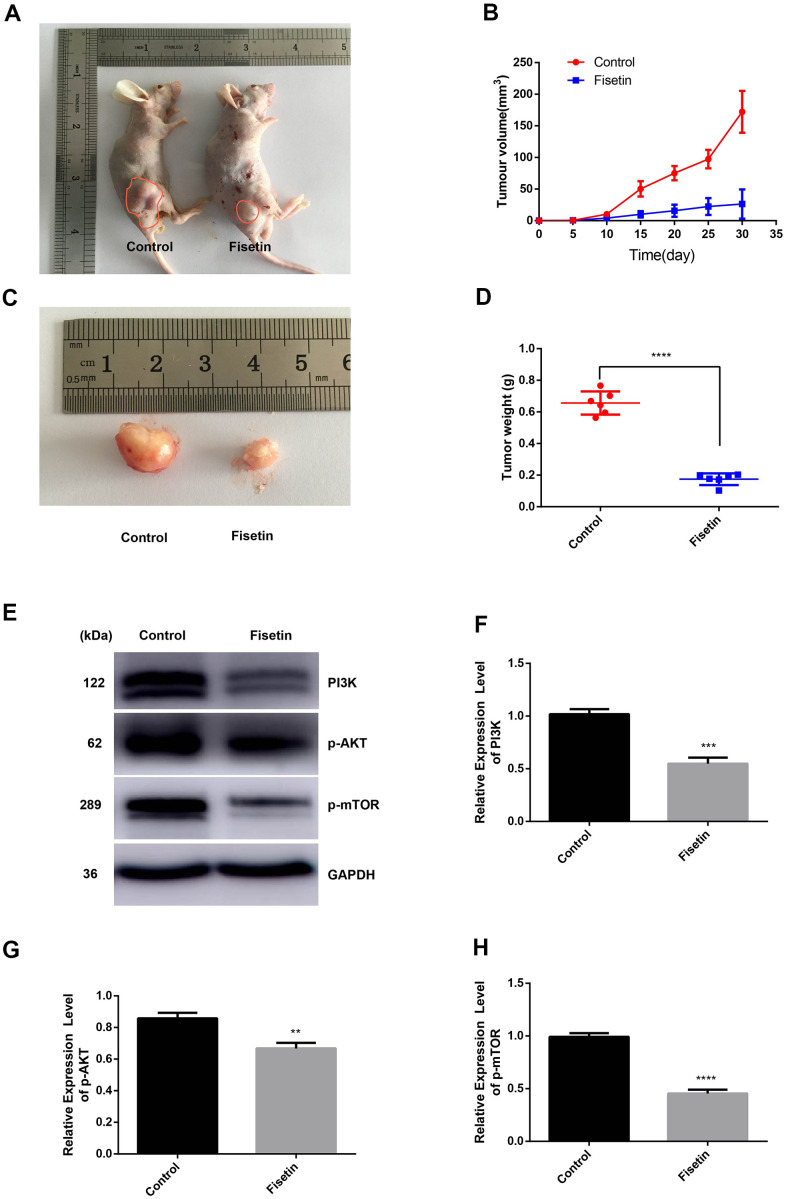
**Fisetin dampens the growth of pancreatic cancer *in vivo*.** (**A**, **C**) Nude mice were subcutaneously inoculated with PANC-1 cells. Following the end of the 30 days, mice along with the tumors were imaged. (**B**) Determination of tumor volume at specified time points; (**D**) Tumors were harvested after 30 days and their weight computed; (**E**) Western blot assessment of protein contents. (**F**) Histogram illustrating PI3K protein contents. (**G**) Histogram illustrating p-AKT protein contents. (**H**) Histogram illustrating p-mTOR protein contents. All experiments were replicated thrice and data are given as means±SD. **P<.01, ***P<.001, ****P<.0001, in contrast with the controls.

Furthermore, to evaluate the effects of fisetin on tumor growth *in vivo*, we conducted a xenografts experiment in nude mice. Figure 8A, 8C illustrates that a remarkable difference in tumor volume was seen as per the tumor image after 30 days. The mean tumor volume along with the weight were remarkably different between the study groups (Figure 8B, 8D). Western blotting illustrated that the expressions of PI3K, p-AKT, as well as p-mTOR proteins were downregulated obviously in fisetin treatment group (Figure 8E, 8F, 8H). These data exhibit that fisetin dampens the pancreatic tumor growth *in vivo*.

## DISCUSSION

Pancreatic cancer is one of the most lethal malignancies globally. Gemcitabine, one of the first-line chemotherapy agents, has successfully enhanced the survival of individuals with diverse cancers, whereas the efficacy of gemcitabine in pancreatic cancer is limited [17]. Therefore, effective agents for treating pancreatic cancer are still needed. Recent investigations revealed that numerous natural products exhibit strong anti-pancreatic cancer effects [18]. For example, baohuoside 1, showed apparent cytotoxicity to two pancreatic cancer cell lines [11]. Fisetin, mainly derived from vegetables and fruits such as cucumber, persimmon, and apple, has been reported to harbor antioxidant, anti-inflammatory, anti-microbial, chemopreventive, chemotherapeutic, and significantly well as anti-cancer activities in recent studies [19–22]. However, studies on fisetin’s effect in pancreatic cancer are unclear, and the possible molecular mechanism of anti-tumor actions has not been elucidated. Herein, we established that fisetin dampens cell growth, migration along infiltration of PANC-1, as well as Patu-8988 pancreatic cells. Besides, a model of xenograft nude mice was adopted to validate the anti-tumor influences of fisetin *in vivo*.

The aberrant growth, migration and invasion capacity of cancer cells needs characteristic changes on numerous key signaling cascades. Janus kinase 2 (JAK2) signaling is a cytokine-stimulated signal transduction cascade which participates in numerous important biological processes, for instance cell growth, differentiation, apoptosis and immune regulation [23]. However, fisetin in this research work exhibited no influences on JAK2 and p-JAK2, but remarkably dampened the phosphorylation of AKT dose dependently in PANC-1 cells (Figure 7). This illustrates that the dampening of pancreatic cancer cells by fisetin targets AKT signaling, rather than JAK2.

Serine/threonine kinase AKT (also termed as protein kinase B or PKB), as a proto-oncogene, has become a significant focus of medical attention because of its remarkable role in modulating diverse cellular functions, consisting of metabolism, transcription, growth, survival, proliferation, along with protein synthesis. Factors can activate axis amplification of AKT signals, consisting of receptor tyrosine kinases, integrins, cytokine receptors, B and T cell receptors along with the G protein-coupled receptors [24, 25]. Herein, we established that fisetin is a specific AKT repressor (Figure 7). Besides, the alterations in PI3K protein contents (upstream protein of AKT), as well as mTOR (direct downstream substrate of AKT) verified the specificity of fisetin on AKT. To summarize, fisetin is a prospective AKT repressor in pancreatic cancer.

As a prospective cancer treatment target, suppression of PI3K/AKT/mTOR cascade could trigger apoptosis [26]. Inhibitors that target the PI3K/AKT/mTOR cascade can improve overall cancer treatment [27]. Recent investigations have documented that PI3K/AKT/mTOR cascade mediates apoptosis of non-small lung cancer [28], esophageal cancer [29], as well as myeloid leukemia cancer [30]. Nonetheless, the precise molecular mechanism of fisetin in pancreatic cancer is unknown. Our data illustrated that apoptosis-linked proteins consisting of cleaved PARP, cleaved caspase 3 and cleaved caspase 8 were upregulated by fisetin dose dependently. Therefore, our data illustrates that fisetin precisely dampens the PI3K/AKT/mTOR cascade through triggering apoptosis in pancreatic cancer. The repression influence of fisetin involves targeting the PI3K/AKT/mTOR axis to activate the Caspases apoptotic cascade. More studies are necessary to determine the bioactive structure of fisetin and the responsive domain of AKT.

## CONCLUSIONS

Herein, we established that fisetin could obviously inhibit the growth, migration, along with the infiltration of pancreatic cancer dose dependently, expanding the anti-cancer class of fisetin. Besides, we determined the possible mechanism of repression by fisetin in pancreatic cancer and discovered that fisetin triggers apoptosis by specifically via targeting PI3K/AKT/mTOR cascade rather than JAK2 signaling. These data suggest that fisetin is a possible, as well as valuable anti-cancer drug for pancreatic cancer and reveal the distinct molecular target of fisetin.
